# Mechanisms of TSC-mediated Control of Synapse Assembly and Axon Guidance

**DOI:** 10.1371/journal.pone.0000375

**Published:** 2007-04-18

**Authors:** Sarah Knox, Hong Ge, Brian D. Dimitroff, Yi Ren, Katie A. Howe, Andrew M. Arsham, Mathew C. Easterday, Thomas P. Neufeld, Michael B. O'Connor, Scott B. Selleck

**Affiliations:** 1 The Developmental Biology Center, Department of Pediatrics, The University of Minnesota, Minneapolis, Minnesota, United States of America; 2 The Developmental Biology Center, Department of Genetics, Cell Biology and Development, The University of Minnesota, Minneapolis, Minnesota, United States of America; Baylor College of Medicine, United States of America

## Abstract

Tuberous sclerosis complex is a dominant genetic disorder produced by mutations in either of two tumor suppressor genes, *TSC1* and *TSC2*; it is characterized by hamartomatous tumors, and is associated with severe neurological and behavioral disturbances. Mutations in *TSC1* or *TSC2* deregulate a conserved growth control pathway that includes Ras homolog enriched in brain (Rheb) and Target of Rapamycin (TOR). To understand the function of this pathway in neural development, we have examined the contributions of multiple components of this pathway in both neuromuscular junction assembly and photoreceptor axon guidance in *Drosophila*. Expression of Rheb in the motoneuron, but not the muscle of the larval neuromuscular junction produced synaptic overgrowth and enhanced synaptic function, while reductions in *Rheb* function compromised synapse development. Synapse growth produced by Rheb is insensitive to rapamycin, an inhibitor of Tor complex 1, and requires *wishful thinking,* a bone morphogenetic protein receptor critical for functional synapse expansion. In the visual system, loss of *Tsc1* in the developing retina disrupted axon guidance independently of cellular growth. Inhibiting Tor complex 1 with rapamycin or eliminating the Tor complex 1 effector, S6 kinase (S6k), did not rescue axon guidance abnormalities of *Tsc1* mosaics, while reductions in Tor function suppressed those phenotypes. These findings show that Tsc-mediated control of axon guidance and synapse assembly occurs via growth-independent signaling mechanisms, and suggest that Tor complex 2, a regulator of actin organization, is critical in these aspects of neuronal development.

## Introduction

Mutations in *TSC1* or *TSC2* result in tuberous sclerosis, a human syndrome characterized by formation of benign tumors, or hamartomas, and a range of neurological and behavioral anomalies, including epilepsy and autism. While neurological dysfunction in patients with tuberous sclerosis is clearly linked to structural brain abnormalities in the central nervous system [1], recent work has provided evidence that TSC1/2 may affect neural development by altering neuronal morphology and function. Loss of TSC function produces changes in dendritic architecture of hippocampal neurons and altered synaptic properties [2]. Rats heterozygous for *TSC2* mutations show disruption of hippocampal physiology, including long term potentiation, a measure of synaptic plasticity [3]. Mutations in the *Drosophila* ortholog of *TSC2, gigas,* have also been shown to produce ectopic axon terminations in addition to the normal projections of sensory neurons [4], [5]. It is unclear to what degree neurological deficits associated with tuberous sclerosis complex result from disruptions of cytoarchitecture in specific brain regions or alterations in synaptic function directly.

*TSC1* and *TSC2* encoded proteins form a complex that regulates a small GTP-binding protein, Ras homolog enriched in brain (Rheb), promoting its endogenous GTPase activity and thereby limiting Rheb signaling. Rheb in turn controls the activity of Target of Rapamycin (TOR), a serine-threonine kinase. The TSC-Rheb-TOR pathway is a critical determinant of growth during development, regulating a number of cellular functions including translation, mRNA turnover, protein stability, and actin organization [6]. It is responsive to growth factors, such as insulin and insulin-like growth factors (ILGFs), and also serves as a nutrient sensor, thus integrating numerous signals related to cell and tissue growth. TOR plays a pivotal role in this signaling pathway, receiving regulatory inputs from Rheb and affecting downstream targets via two distinct molecular complexes. Tor complex 1 (TORC1) includes Raptor and mLST8, and regulates translation via phosphorylation of S6 kinase (S6K) and 4E-binding protein (4EBP). Tor complex 2 (TORC2) includes Rictor in addition to Tor and mLST8; in both yeast and mammalian cells TORC2 influences the actin cytoskeleton. Tor complex 1, but not Tor complex 2, is inhibited by the anti-proliferative and immunosuppressant compound rapamycin, emphasizing that TORC1 and 2 are pharmacologically separable entities. The distinct molecular outputs of TORC1 and 2 have also suggested that TORC2 may be the primary regulator of cell polarity and morphology. It is not known which functions of TSC-Rheb-TOR in the nervous system are mediated by either or both of the two Tor kinase-containing complexes, and if pharmacological intervention in tuberous sclerosis complex patients should best be directed at TORC1, with agents such as rapamycin, or if TORC2-specific agents will also be important.

The fruit fly *Drosophila* has proven to be an important system for understanding the molecular mechanisms of Tsc-Rheb-Tor signaling during development [7]. As in vertebrates, this signaling cascade is a critical regulator of growth. All of the principal elements of this pathway are represented in *Drosophila*, including molecular components upstream of Tsc, such as phosphatidyinositol-3 kinase (Pi3K), Akt, Pten and the insulin receptor ortholog, InR. Likewise, molecules that convey the signal downstream of Tsc, including Rheb, Tor, and S6k serve critical roles in the fruit fly. Mutations affecting all these genes have been identified in *Drosophila*, as well as transgenes that can convey gain-of-function effects. We have used these molecular and genetic tools to explore the function of Tsc-Rheb-Tor signaling in two fundamental processes essential to nervous system development, synapse formation and axon guidance.

The *Drosophila* neuromuscular junction has served as a powerful model for identifying the molecular components required for assembly and plasticity of a defined synapse [8]. This glutamatergic synapse must respond to greater than a 100-fold increase in the size of the muscle target from first to third instar larval stages. Physiological responses of this synapse are well-characterized using single-cell recording techniques, and morphological development with specific molecular markers has been extensively described. We have used this synapse to determine the role of gain or loss of Tsc-Rheb-Tor signaling on synapse assembly and function.

The visual system of *Drosophila* is equally well described in both molecular and genetic terms [9]. Photoreceptors show stereotyped projections to the brain, and genes required for photoreceptor axon projection and termination have been identified in numerous screens. Methods for making somatic cell mosaics have proven particularly powerful in determining what molecules are required in photoreceptors or in cells along their trajectory into the brain. Previous studies have shown that retinal clones mutant for the *Drosophila* Tsc2 ortholog *gigas* generated enlarged ommatidia with increased numbers of synaptic contact sites in the optic lamina [10]. We have taken advantage of this system to examine Tsc-Rheb-Tor requirements for photoreceptor axon guidance and formation of functional synaptic contacts in the brain.

Our results establish that either gain or loss of signaling via the Tsc-Rheb-Tor pathway affects synapse development at the *Drosophila* NMJ. Ectopic activation of the Tsc-Rheb-Tor signaling pathway produced profound synaptic overgrowth with commensurate increases in synaptic function. We show that Rheb-mediated enhancement of synaptic function depends upon bone morphogenetic protein (BMP) signaling mediated by *wishful thinking (wit)*, a type II receptor. In the visual system, increased Tsc-Rheb-Tor signaling produced cell autonomous defects in photoreceptor axon guidance. Both genetic and pharmacological evidence suggest that TORC2 serves critical functions in both synapse development and axon guidance in *Drosophila*. Axon guidance phenotypes produced by null mutations in *Pten* and *Tsc1* are distinct, demonstrating that regulation of signaling by these two tumor suppressor genes are not functionally equivalent in the nervous system.

## Results

### Activation of Tor signaling produces synaptic growth and enhanced synaptic function

**Figure 1:**
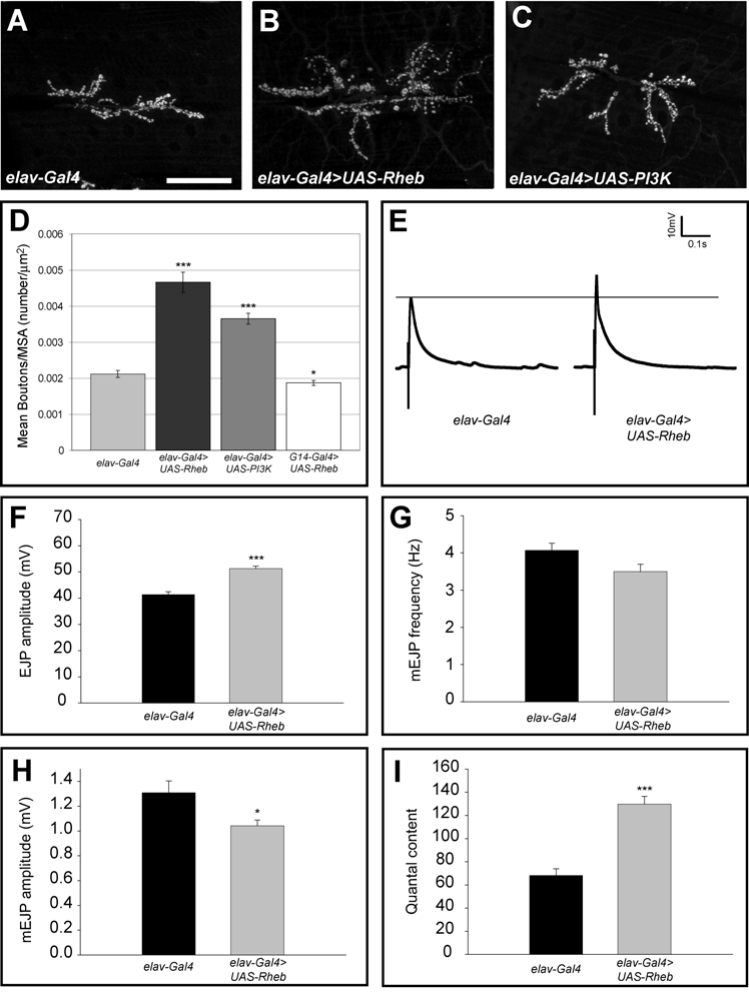
Activation of the Tor pathway produces synaptic growth and enhanced physiological function. The morphology of the third instar larval NMJ was visualized with the presynaptic marker anti-cysteine string protein (CSP) and confocal microscopy. Images shown are stacks of 20 or more optical sections. Neuronal (*elav-Gal4*) expression of either *Rheb* (B) or *Pi3K* (C) increased the size of the synapse compared to control animals bearing the *elav-Gal4* transgene alone (A). Numbers of synaptic boutons/muscle area are quantified in D. Expression of either *UAS-Rheb* (n = 41) or *UAS-Pi3K* (n = 41) produced a significant increase in the number of boutons/muscle area compared to controls (n = 44), while expression of *UAS-Rheb* in the muscle (driven by *G14-Gal4*, n = 18) produced a reduction. Neuron-specific expression of *Rheb* also produced electrophysiological changes at the NMJ, determined by intracellular recordings from abdominal muscle 6 in third instar larvae. The amplitude of the EJP was significantly increased in animals expressing *UAS-Rheb* (n = 21) compared to controls with *elav-Gal4* alone (n = 12). Examples of EJP voltage traces are shown in E, and mean EJP values are quantified in F. Quantal content, a measure of the number of synaptic quanta released in a single firing of the motoneuron, was nearly doubled by neuron-directed expression of *Rheb* compared to controls (I). Mini-EJP amplitude was decreased in these animals (H), while mEJP frequency showed no significant change (G). In this and all subsequent figures, *** denotes p-values less than 0.00005 using a student t-test comparison with controls, ** denotes p-values less than 0.005, and * denotes p-values less than 0.05. The scale bar is 50 microns in panel A.

Tsc1/2 affect growth by inhibiting Rheb, a small GTP-binding protein that in turn governs Tor activity. Overexpression of Rheb activates the pathway independent of *Tsc* gene function [11]–[13]. We have used the *Gal4-UAS* system to overexpress *Rheb* in either the motoneuron or the muscle of the *Drosophila* third instar larval neuromuscular junction (NMJ), a well-characterized glutamatergic synapse [8] that shows dynamic growth during larval development. Ectopic expression of *Rheb* in the motoneuron of the third instar larval NMJ using a pan-neuronal *Gal4* line (*elav-Gal4*) resulted in more than a doubling of synapse size, measured by the number of synaptic boutons/muscle area (Figure 1A–C, quantified in D). Similar results were seen using a motoneuron-specific *OK6-Gal4* line (data not shown). We found no evidence of motoneuron axon misrouting at this level of Rheb activation; the motoneuron axon follows the normal trajectory and synapses at the correct location on muscle 6 and 7(data not shown). Indeed, *elav-Gal4>UAS-Rheb* animals are viable, indicating this degree of pathway activation is considerably more mild than loss of *Tsc1* (see below). Expression of Rheb selectively in muscle (*G14-Gal4>UAS-Rheb*), while producing enlargement of muscle cells, did not increase the proportional size of the synapse (bouton number/muscle area, Figure 1D). Activation of Tor by overexpression of Pi3K in the motoneuron also produced an enlarged synapse, but to a lesser degree than overexpression of Rheb (Figure 1C, D).

Enlargement of the NMJ in *Drosophila* is not always associated with an electrophysiologically competent synapse. For example, *highwire* mutants display large NMJs but markedly compromised synaptic function [14], [15]. We therefore assessed the electrophysiological behavior of the NMJ in animals overexpressing Rheb in the motoneuron. This synapse showed nearly a doubling of the quantal content, a measure of the number of synaptic vesicles released per motoneuron firing (Fig 1I). The amplitude of the excitatory junctional potential (EJP), the voltage change in the muscle elicited by a suprathreshold stimulation of the motoneuron, also increased significantly compared to control synapses (Figure 1E, F). Mini-excitatory junctional potentials (mEJPs) are depolarizations of the muscle that result from spontaneous neurotransmitter release and provide a measure of vesicular fusion. While the mEJP frequencies of *Rheb* overexpressing animals showed no significant change (Figure 1G), the mEJP amplitudes were lower than matched controls (Figure 1H). In all, activation of Tor signaling via overexpression of Rheb produced an expanded synapse that was fully functional.

### Reduction of Tor signaling produces a small synapse with compromised function

**Figure 2:**
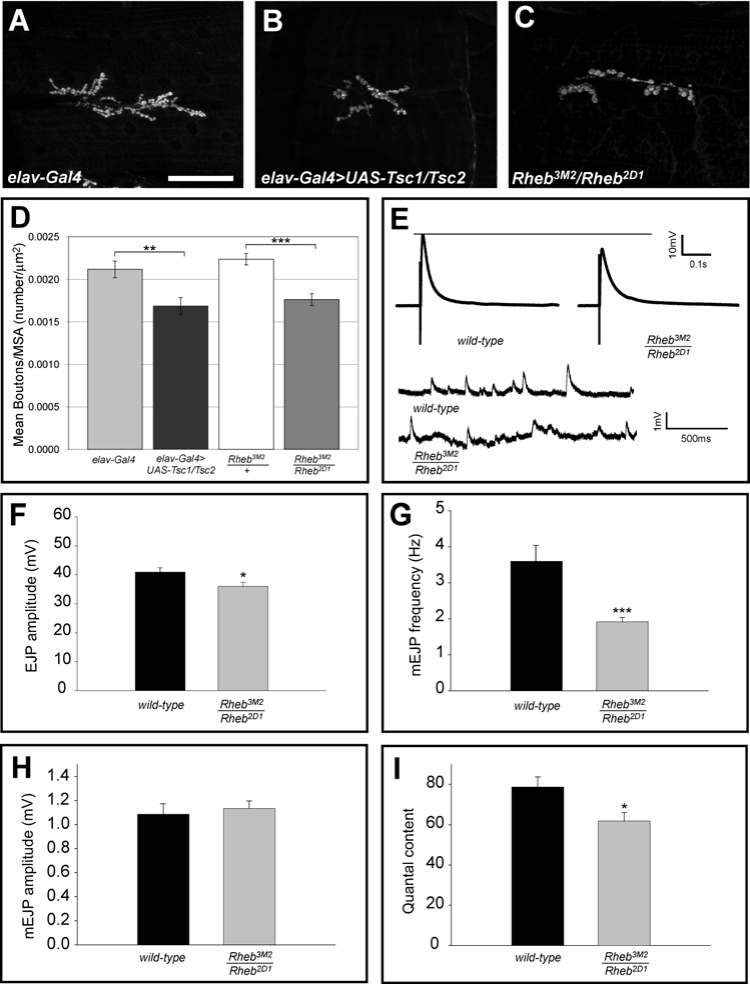
*Rheb* activity is required for normal synapse assembly. Panels A–C show anti-CSP staining of larval NMJs in a control animal (A, *elav-Gal4* driver alone), an animal bearing *elav-Gal4>UAS-Tsc1, UAS-Tsc2* (B), or a *Rheb* partial loss of function mutant (C). Reduction of *Rheb* function produced by either neuron-directed expression of *Tsc1* and *Tsc2* (n = 22) or mutation of *Rheb* (n = 40) significantly reduced synapse size compared to controls with *elav-Gal4* alone (n = 44) or animals heterozygous for a *Rheb* mutation (n = 17), as measured by the number of synaptic boutons/muscle area (D). Panel E shows sample EJP traces for wild-type and *Rheb* mutant NMJs, as well as baseline recordings from these preparations showing the size and frequency of mini-EJPs. Panels F, G, and I show reductions in EJP amplitude, mini-EJP frequency, and quantal content for *Rheb* mutant synapses (n = 29) compared to wild-type controls (n = 10). Mini-EJP amplitude did not show a significant change (H). The scale bar in A is 50 microns.

To determine if reduced Tsc-Rheb-Tor signaling compromises synapse growth and function we overexpressed Tsc1 and Tsc2 in the motoneuron, or compromised *Rheb* activity using a combination of hypomorphic *Rheb* alleles previously shown to cause reductions in cell size and number as well as S6k activity [11]. Overexpression of *UAS-Tsc1/Tsc2* has been shown to limit growth mediated by Rheb [11]–[13], and we observed that Tsc1 and 2 overexpression in the motoneuron reduced synapse size compared to controls (Figure 2A, B, quantified in D). Consistent with this finding, *Rheb* hypomorphic mutant larvae showed a significantly reduced number of boutons per unit muscle area compared to heterozygous controls (Fig 2C, D). The NMJs of these animals also revealed significant changes in synaptic function. mEJP frequencies in *Rheb* mutant animals were half that of controls (Fig 2E, G), and EJP amplitudes were significantly reduced (Figure 2F). We also saw a reduction in the quantal content of *Rheb* mutants (Figure 2I), while mEJP amplitude showed no significant change (Figure 2H). Thus, reducing Tor activity by either of two mechanisms, overexpression of Tsc1/2 or partial loss-of-function mutations in *Rheb*, compromised synapse morphological development and function. Electrophysiology of hypomorphic *Tor* mutants showed a reduction in mEJP frequency similar to what we saw for Rheb mutants (data not shown).

### Rapamycin does not inhibit synapse growth mediated by overexpression of Rheb

**Figure 3:**
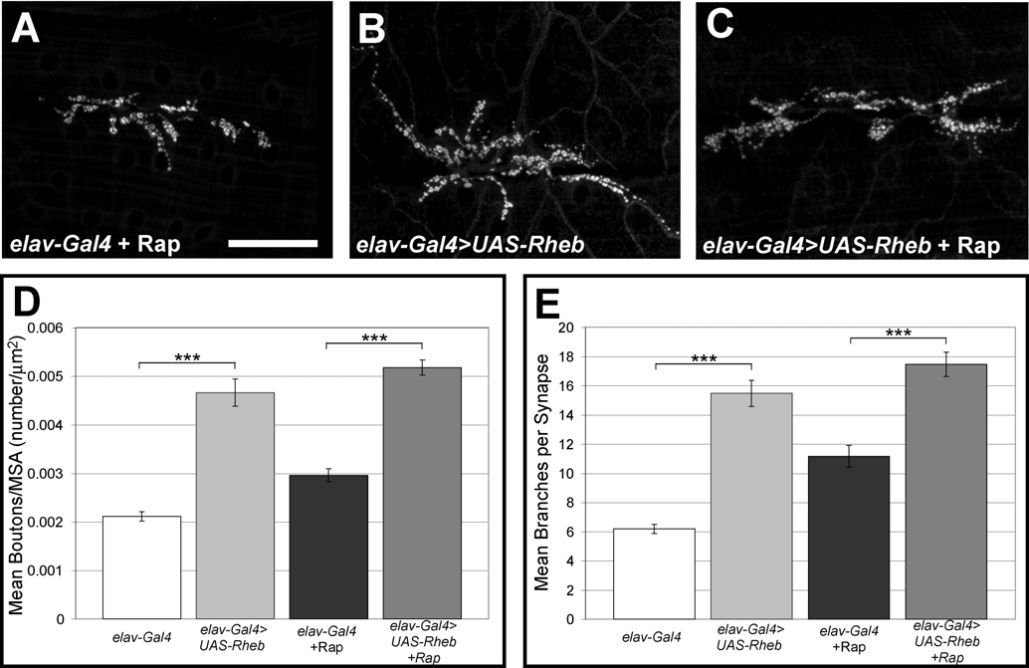
Rapamycin does not block *Rheb-*mediated synapse growth. Panels A–C show anti-CSP staining of NMJ synaptic boutons, demonstrating that the TORC1 inhibitor rapamycin does not block synapse growth in control animals or in larvae with neuron-directed expression of *Rheb* (*elav-Gal4>UAS-Rheb*). Panels D and E provide quantification of bouton numbers/muscle area and numbers of motoneuron branches, respectively, for *elav-gal4* controls (n = 44), animals with neuronal expression of *Rheb* (n = 41), control animals treated with rapamycin (n = 26), and *Rheb* expressing animals treated with rapamycin (n = 29). The scale bar is 50 microns.

To evaluate if Rheb overexpression-mediated synapse expansion takes place via known growth regulatory pathways, we grew larvae from hatching to the third instar larval stage on rapamycin-containing food. Rapamycin has been shown to block growth mediated by TORC1 in *Drosophila*, and we used a concentration that produced clear developmental delay [16]. Culturing larvae bearing *elav-Gal4* and *UAS-Rheb^+^* transgenes on rapamycin reduced overall growth, including muscle size, but did not suppress the synaptic enlargement measured either by the number of synaptic boutons/muscle area or the number of motoneuron branches (Figure 3A–C, quantified in D, E). These findings show that Rheb-mediated synaptic growth did not require TORC1 activity, implicating TORC2 and its regulation of the actin cytoskeleton as serving critical functions in synaptic growth control. Interestingly, culturing *elav-Gal4* control larvae (without the *UAS-Rheb* transgene) on rapamycin produced both an increase in the number of boutons per unit muscle area and an in increase in motoneuron branching (Figure 3D, E). This raises the possibility that blocking the activity of TORC1 with rapamycin indirectly influences the activity of other Tor complexes.

The Tsc-Rheb-Tor pathway regulates translation largely by controlling S6k [11]–[13]. Rheb activation of Tor produces phosphorylation and activation of S6k. The control of translation via S6k represents but one component of regulation affected by this pathway and is molecularly distinct from Tsc-Rheb-Tor-mediated control of the actin cytoskeleton. To evaluate the contribution of S6k to synapse growth we examined the NMJs of animals bearing a null mutation in *S6k. S6k* mutants are small, with a reduced muscle surface area compared to controls. The synapse size, however, as measured by the number of boutons per unit muscle area, is not reduced (data not shown). These findings contrast with the effects of *Rheb* mutations or *Tsc1* and *Tsc2* overexpression (Figure 2), but are consistent with the finding that rapamycin does not suppress synapse overgrowth (Figure 3). Together, these results suggest that TORC1 and S6k do not contribute significantly to the proportional growth of the NMJ.

### Rheb-mediated changes in synapse function require the BMP-signaling receptor encoded by *wishful thinking*

**Figure 4:**
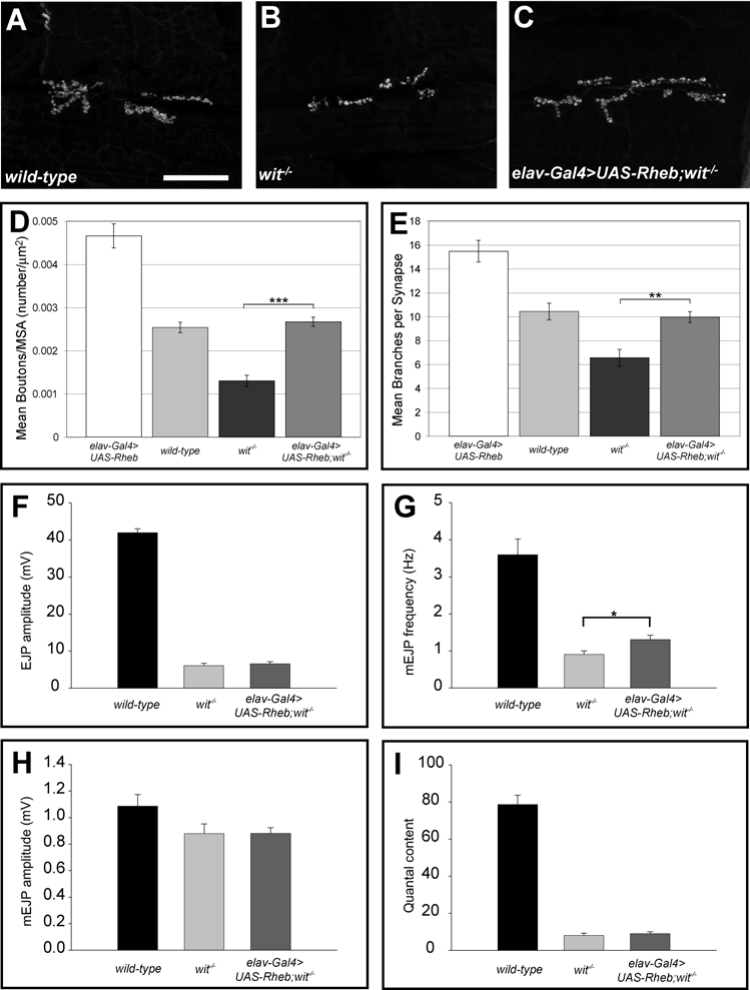
*Rheb*-mediated synapse expansion and physiological function is BMP-signaling dependent. Anti-CSP staining of synaptic boutons (panels A–C) shows the effects of *wit* on synapse growth (B), and the effects of neuron-directed expression of *Rheb* on *wit* mutant NMJs (C) compared to wild-type (A). Synapse size, measured by either the number of boutons/muscle area (D) or the number of motoneuron branches (E), is dramatically reduced in *wit* mutants (n = 20) compared to wild-type (n = 12), and is partially rescued by neuron-directed expression of *Rheb* (*elav-Gal4>UAS-Rheb*, n = 24). Reductions in EJP amplitudes (F), mini-EJP amplitudes (H), and quantal content (I) mediated by loss of *wit* (n = 8) are not rescued by neuron-directed expression of *Rheb* (n = 16) (n = 10 for wild-type)*.* The decrease in mini-EJP frequency of *wit* mutants, a measure of spontaneous vesicle release, is rescued to a significant degree by expression of Rheb in the motoneuron (G). The scale bar represents 50 microns.

Growth factor-mediated signaling, including both Wingless (Wg) and BMP pathways, is important for normal NMJ growth in *Drosophila*. Animals bearing mutations in *wishful thinking (wit)*, a gene encoding a BMP type II receptor, show a very small NMJ with dramatically compromised synaptic function [17]–[19]. To determine the relationship between Rheb-regulated synaptic growth and BMP-mediated synapse assembly we tested the ability of Rheb overexpression to rescue the synaptic growth defect of *wit* mutants. While *Rheb^+^* expression in the motoneuron was able to restore the number of synaptic boutons to wild-type levels in *wit* mutant larvae, the number of boutons was significantly less than with Rheb overexpression alone (Figure 4A–E). Rheb overexpression modestly increased mini EJP frequency of *wit* mutants (Figure 4G), but showed no capacity to rescue either quantal content or EJP amplitudes (Figure 4F–I), therefore *wit* is clearly required for most Rheb-directed effects on synapse function. While these results do not establish the nature of the communication between Tor-Tsc signaling and the BMP pathway, it does demonstrate that an intact BMP system is necessary for Rheb-directed changes in synapse function.

### Tsc-Rheb-Tor signaling is critical for axon guidance in the visual system

**Figure 5:**
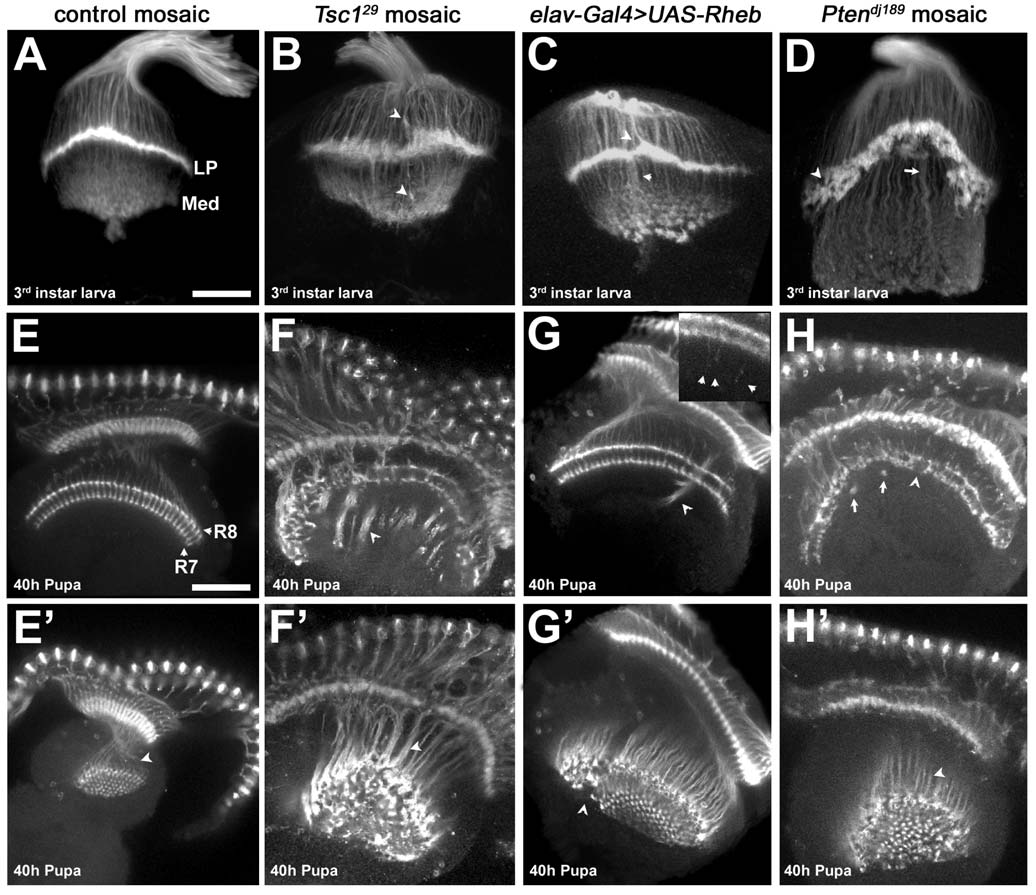
Photoreceptor axon projection defects associated with increased Tor signaling. (A–D) Dorsal-posterior views of third instar optic lobes stained with MAb24B10 to visualize photoreceptor projections. (A) Mitotic clones in an *FRT82B* control background show proper termination of photoreceptor axons R1-6 at the lamina plexus (LP), and termination of photoreceptors R7 and R8 in the medulla (Med). (B) *Tsc1^29^* mutant axons terminate at incorrect positions above and below the lamina (arrowheads) and produce a broadened lamina plexus. (C) Neuronal expression of Rheb creates axon termination defects similar to those seen in *Tsc1* mosaics (D) *Pten^dj189^* mutant photoreceptors leave gaps and holes (arrowhead) in the lamina plexus, which is broader and noticeably “peaked.” The medulla contains axon projections which are thicker and much longer than in controls (arrow). (E–H′) Dorsal view of optic lobes from 40h pupae stained with MAb24B10. E′–H′ are lower optical planes of the optic lobes shown in E–H, respectively. (E, E′) Control photoreceptors R7 and R8 show two distinct layers of termination in the medulla (labels), and are arranged in a highly regular pattern (arrowhead). (F) Animals with *Tsc1^29^* mutant photoreceptors show severe disruption of the R7 and R8 termination layers. Instead of terminating at the correct positions, the axons fail to de-fasciculate, forming dense bundles (arrowheads) that project beyond the medulla. (G, G′) Neuron-directed expression of Rheb causes axon bundles to project beyond the medulla in a fashion similar to *Tsc1* mosaics (arrowheads), but the phenotype is much less severe. (G, inset) Individual Rheb-overexpressing axons show an intermediate termination defect, stopping several microns beyond their normal targets (arrowheads in inset). (H) *Pten^dj189^* mutant axons exhibit gaps and collapses in the R7/R8 termination zone (arrowhead). Thick axon bundles can be seen that bypass their usual stopping points and then loop back to terminate at other locations in the R7/R8 layers (arrows). (H′, F′) Axon bundles in *Pten^dj189^* mosaics are not as densely packed as those of *Tsc1^29^* mosaics (arrowheads), but are still disorganized. All scale bars are 50 microns.

Another fundamental aspect of neural development is the correct specification of axon pathfinding and synapse formation with the correct targets. The *Drosophila* visual system offers a powerful experimental model for assessing the function of a signaling system in axon guidance. To evaluate the function of the Tsc-Rheb-Tor signaling pathway in axon guidance we generated genetic mosaic animals where mutant photoreceptor neurons project to a phenotypically wild-type brain. In the fruit fly *Drosophila,* each retinal sensory unit, or ommatidium, is comprised of eight photoreceptors, R1-8. In the third instar larval brain, R1-6 project to the first optic ganglion, the lamina, and terminate to form a discrete plexus where synapses will form later in development (Figure 5A). R7 and R8 project to a deeper level in the brain, the medulla, forming a discrete set of projections seen in both larval and pupal brains. In 40h pupae the R7 and R8 projections terminate in distinct layers in the medulla, producing a highly regular and stereotyped pattern (Figure 5E). Loss of *Tsc1* function in the retina produces an enlarged eye disc with an increased number of photoreceptors [20]–[22]. Axon projections from *Tsc1* mutant photoreceptors in the brains of third instar larvae and pupae showed severe axon guidance abnormalities (Figure 5B, F, F′, quantified in Table 1). In third instar larvae, R1-6 termination at the lamina plexus is disorganized, producing an irregular termination zone (compare Figure 5A to 5B). R7/R8 terminations within the larval medulla are also abnormal (Figure 5B, arrowhead). At the 40 hr pupal stage we observed gaps in the R7/R8 layers with large axon bundles, or fascicles, that projected past their appropriate termination points (Figure 5F, F′).

**Table 1:** Axon guidance defects in animals with altered Tsc-Rheb-Tor signaling

(Percent of optic lobes affected)								
						Incorrect Terminations		
3^rd^ instar larvae	Thick LP	Gaps in LP	LP Peaked	Long R7/8	Gaps in Med.	Above LP	Below LP	In Med.
*Tsc1^29^* (n = 58)	70	41	7	5	31	45	52	72
*Pten^dj189^* (n = 38)	24	100	79	63	29	58	68	95
*Rheb^3M2/26.2^* (n = 22)	68	55	0	0	41	32	18	36
*Tor^A948V^* (n = 12)	17	17	0	8	0	0	17	33
*S6k^l-1^* (n = 49)	29	41	2	0	2	10	14	4
*Tor^A948V^ Tsc1^29^* (n = 14)	21	29	0	0	7	7	0	0
*S6k^l-1^ Tsc1^29^* (n = 23)	65	83	0	0	70	43	17	43
wild-type +Rap (n = 80)	19	13	0	0	9	21	14	13
*Tsc1^29^* +Rap (n = 60)	76	54	2	6	59	46	71	63
**40-hour pupae**		**Pathfinding Defects**		**De-fasciculation Defects**		**Termination Layer Defects**		
*Tsc1^29^* (n = 60)		100		100		100		
*elav-Gal4>UAS-Rheb* (n = 23)		83		43		39		
*Pten^dj189^* (n = 73)		25		30		8		
*Rheb^26.2^* (n = 80)		36		3		19		
*S6k^l-1^* (n = 32)		28		9		25		
*Tor^A948V^* (n = 20)		35		0		10		
*Tor^A948V^ Tsc1^29^* (n = 25)		40		4		16		

*Tsc1^29^, Pten^dj189^, and *Rheb^26.2^* are *eyFLP* mosaics; all others are mutants. **LP** - lamina plexus; **Med.** - medulla; **Rap** - rapamycin

To evaluate the degree of pathway activation mediated by pan-neuronal expression of *UAS-Rheb*, which we used above to evaluate the role of Tor signaling at the NMJ, we examined photoreceptor pathfinding in *elav-Gal4>UAS-Rheb* larvae and pupae. These animals survive to adulthood and show disruptions in photoreceptor projections, but to a significantly lesser extent than found in *Tsc1* mosaic animals (see Table 1). In *elav-Gal4>UAS-Rheb* pupal brains, abnormal bundles of axons that penetrate into deeper brain structures were found, but this phenotype was markedly less severe than in *Tsc1* mosaic animals (Figure 5G, G′, Table 1). Close inspection of R7 and R8 endings in the medulla revealed individual photoreceptor axons growing past the correct termination site (Figure 5G inset). R1-6 endings in the larval brain also show irregularities, but the lamina plexus is less disrupted than in *Tsc1* mosaics (Figure 5C). These findings indicate that the degree of pathway activation achieved with *elav-Gal4>UAS-Rheb* is markedly less than produced by loss of *Tsc1*. Moreover, these results suggest that there is a continuum of axon pathfinding abnormalities with different levels of pathway activation.

*Pten*, another negative regulator of cell growth and proliferation, encodes a phosphatase that converts the lipid signaling molecule phosphatidylinositol 3,4,5 triphosphate (PIP_3_) to PIP_2_, an inactive form, thus antagonizing PI3K activation of the TOR pathway. Like *Tsc1, Pten* retinal mosaics show eye overgrowth and precocious differentiation [23]–[27]. To determine if disruptions of *Pten* function affect axon guidance, we generated mosaic animals. *Pten* mutant photoreceptor projections showed disorganization of axon termini in the third instar larval brain and were notable for a misshapen and concave lamina plexus with a large number of gaps (Figure 5D, quantified in Table 1). At the pupal stage, *Pten* mutant projections showed significantly less severe defects than photoreceptors bearing a *Tsc1* null mutation (Figure 5H, H′ and Table 1), with fewer projections failing to stop at the normal medulla termination sites. The penetrance of pathfinding, defasciculation, and termination defects in 40h pupae was lower in *Pten* than in *Tsc1* null mutant photoreceptors projecting to a wild-type CNS (Table 1). In sum, *Pten* and *Tsc1* mutant photoreceptor projections show distinct patterns of photoreceptor axon guidance defects, despite the fact that these two inhibitors of Tsc-Rheb-Tor signaling have similar influences on cell size, growth, and differentiation [20]–[22], [24]–[26], [28].

We also observed distinct effects of *Tsc1* and *Pten* retinal mosaics on the differentiation of lamina neurons and visual system glia, detected with anti-Dachshund and anti-Repo antibodies, respectively (Figure S1). *Pten* mutant retinal projections produced an abnormally large lamina not seen in *Tsc1* mosaics (Figure S1A–C). In both *Pten* and *Tsc1* mosaics visual system glia were found in the brain in roughly normal positions (Figure S1D–F), although some disorganization was evident in brains receiving *Tsc1* mutant photoreceptor projections. It is possible that this disruption of glial architecture may partially contribute to the axon projection defects observed in *Tsc1* mutants.

**Figure 6:**
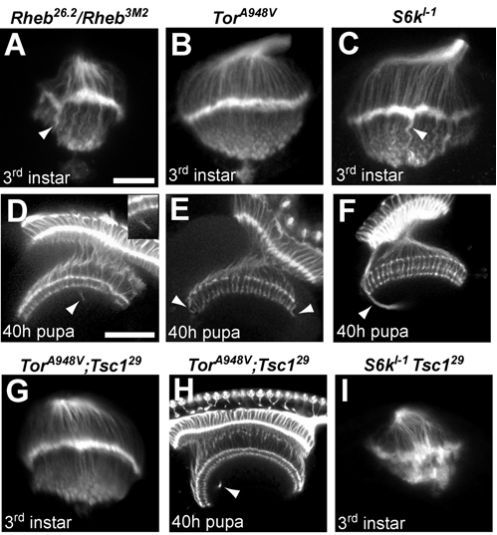
Effects of mutations that downregulate the Tor pathway on photoreceptor axon guidance, and genetic epistasis with *Tsc1*. Optic lobes from third instar larvae (A–C) and 40h pupae (D–F) stained with MAb24B10. (A) Larvae heteroallelic for a hypomorphic combination of *Rheb* alleles show abnormal photoreceptor patterning and contain thick axon bundles that extend into the medulla (arrowhead). (D) At the 40 h pupal stage, *Rheb* mutants display axons that bypass their normal targets in the R7/R8 termination zones (arrowhead). (B) Larvae homozygous for a hypomorphic *Tor* allele show fairly normal photoreceptor patterning, but at the pupal stage (E) misrouted axons can be seen in the medulla (arrowheads). (C) *S6k* null homozygous larvae show thick axon bundles projecting past the lamina (arrowhead), while *S6k* pupae (F) display misrouted axons that initially bypass the R7/R8 termination zone (arrowhead). (G, H) Animals doubly mutant for *Tor* and *Tsc1* do not show the severe photoreceptor defects seen when axons are mutant for *Tsc1* alone (compare to Figure 5B, F, F′), although mild defects similar to those in *Tor* mutants are still apparent (arrowhead). (I) *S6k-Tsc1* double homozygous mutants display a severe phenotype dissimilar to mutants for either *S6k* or *Tsc1* alone. The scale bar is 25 microns in panel A, 50 microns in panel D.

To evaluate the effects of reduced Tor signaling, we examined axon guidance in animals bearing hypomorphic mutations in *Tor* and *Rheb*, as well as a null allele of *S6k*, a key downstream target of TORC1. In all three of these mutants, mild axon projection defects were observed (Figure 6A–F, Table 1). Third instar larvae had irregular laminas and abnormally thick projections to the medulla (Figure 6A–C, arrowheads). In 40 h pupae, R7 and R8 terminations were largely normal, but there were projections which misrouted and failed to terminate correctly (Figure 6D–F, Table 1). Genetic mosaic analysis of *Rheb* mutant photoreceptor projections showed the same phenotypes, demonstrating that normal levels of Tor-Tsc signaling in the retina are required for proper photoreceptor targeting (data not shown). These findings establish that reductions in Tor-Tsc signaling also produce axon guidance defects, although quite mild in comparison to activation of the pathway achieved by loss of *Tsc1* function. However, only the *S6k* mutants are null in these experiments, and we cannot therefore fully assess the contributions of *Tor* or *Rheb* to axon guidance compared to *Tsc1*.

To determine if the functional relationships critical for growth control are also in effect for axon guidance, we conducted genetic epistasis experiments between *Tsc1* and both *Tor* and *S6k. Tsc1* mosaic pupae show severe axon guidance abnormalities and *Tsc1* mutant animals do not survive to the pupal stage; in contrast, animals bearing both a *Tsc1* mutation and a hypomorphic *Tor* allele survived to pupal stages and showed only modest axon guidance abnormalities in larval and pupal brains (Figure 6G, H, Table 1). The gross disruptions of R7/R8 terminations in the medullas of 40h *Tsc1* mosaic pupae were almost completely rescued by the presence of a hypomorphic allele of *Tor*. Genetic mosaics with *Tsc1 Rheb* double mutant chromosomes also showed dramatic rescue of photoreceptor axon guidance defects (data not shown). In contrast, *S6k* null mutations did not ameliorate the *Tsc1* axon projection defects in the larval brain, and both the lamina plexus and medulla projections were highly disordered (Figure 6I, Table 1). These findings demonstrate that Tor and Rheb, but not S6k, are critical components of the photoreceptor axon guidance signaling system downstream of Tsc1.

**Figure 7:**
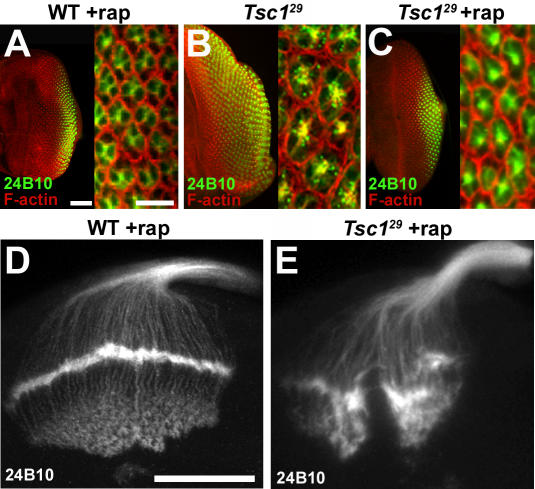
Axon guidance defects in *Tsc1* mosaics are not suppressed by blocking growth. (A–C) Third instar eye discs from wild type and *Tsc1* mosaic larvae raised with or without rapamycin (rap). Ommatidial units, comprised of eight photoreceptors, were visualized with phalloidin (red) that detects F-actin, and MAb24B10 (green). Phalloidin staining is strongest at the perimeter of each ommatidium, outlining each sensory unit. Rapamycin treatment of *Tsc1* mosaic eye discs (C) restored eye disk size and cell size compared to wild type (A). (D and E) Rapamycin treated third instar larval brains stained with MAb24B10. Rapamycin treatment blocked abnormal growth of the retina and the increase in photoreceptor cell size, but did not ameliorate the abnormal axon projections also characteristic to untreated *Tsc1^29^* mosaics. The scale bars in panel A represent 50 microns in the left image, 10 microns in the right image. The scale bar is 50 microns in panel D.

In order to evaluate if the growth control functions of Tsc-Rheb-Tor signaling are important for axon guidance, we used rapamycin to inhibit the abnormal growth produced by loss of *Tsc1* function. Feeding animals with rapamycin between hatching and the third instar larval stage blocked the retinal cell growth and proliferation defects of *Tsc1* mutant photoreceptor mosaics. This was evident in both the overall size of the developing retina and the size of the photoreceptor cell bodies (Figure 7A–C). While the growth defects of *Tsc1* mosaics were rescued by rapamycin treatment, photoreceptors from these animals still showed severe axon guidance abnormalities in the third instar larval brain, with an irregular and disrupted lamina plexus, as well as disorganized projections to the medulla (Figure 7E, Table 1). Treatment of wild-type controls with rapamycin produced only mild defects in the lamina plexus (Figure 7D, Table 1) supporting the hypothesis that Tsc1-mediated regulation of axon guidance operates largely via a rapamycin-insensitive function of Tor. We noted that the excessive growth of *Pten* mutant retinas was not rescued by rapamycin treatment, in contrast to the effects of this TORC1 inhibitor on *Tsc1* mosaics. While the growth and differentiation phenotypes of *Pten* and *Tsc1* mutant retinas are comparable, the difference in their rapamycin responses highlights how disruption of signaling by these two regulators is distinct.

## Discussion

### Tsc-Rheb-Tor signaling in neural development

The Tsc-Rheb-Tor pathway is critical for integrating a variety of signals that govern cellular and organismal growth. Inappropriate activation of the pathway also leads to severe neurological and behavioral abnormalities, including mental retardation, autism, and epilepsy [1], [6]. While *TSC* mutations produce hamartomatous growths in the brain, recent evidence has suggested that these benign tumors may not be solely responsible for the nervous system dysfunction that is a hallmark of tuberous sclerosis complex. Loss of *TSC2* in hippocampal neurons produces changes in neuronal morphology and synaptic transmission [2]. Heterozygosity for *TSC2* in the rat compromises several measures of hippocampal long term potentiation [3]. Loss of *Pten*, an important upstream regulator of Tsc-Rheb-Tor signaling, in a limited set of neurons also affects neuronal morphology and socialization behavior [29]. These findings collectively provide evidence that Tsc-Rheb-Tor signaling is critical for the morphological and functional development of the nervous system. It is not clear, however, if the entire Tsc-Rheb-Tor signaling network is critical for nervous system development, or if neural function is strictly a consequence of altered growth regulation. It is also not known if loss of signaling is as detrimental to neuronal development as inappropriately elevated signaling, such as occurs with loss of *TSC* function. We have taken advantage of the genetic and molecular tools available in the fruit fly *Drosophila* to address these questions. Our findings demonstrate that appropriate levels of Tsc-Rheb-Tor signaling are critical for both NMJ development and for axon guidance in the visual system. In both these contexts, effects are independent of growth, implicating TORC2 rather than TORC1 as the complex mediating Tsc-Rheb-Tor signaling influences in the nervous system.

### Tsc-Rheb-Tor effects on neural development are independent of growth regulation

Given the importance of Tsc-Rheb-Tor signaling in regulating cellular and tissue growth, it was important to determine if disruption of this pathway affects neural development via its effects on growth or through signaling components independent of those that govern cellular size and growth. To address this issue we used both pharmacological and genetic methods to block the increased growth produced by pathway activation. The immunosuppressant rapamycin is a TORC1-specific inhibitor that prevents activation of S6k and blocks growth mediated by loss of *Tsc1*. Rapamycin treatment retarded growth in larvae with pan-neuronal expression of *Rheb*, but failed to reduce the synapse expansion characteristic of these animals. Similarly, while rapamycin effectively reduced the retinal overgrowth of *Tsc1* mosaic animals, it failed to suppress the photoreceptor axon guidance defects seen in the visual system. Loss of *S6k* function also failed to ameliorate axon guidance defects in *Tsc1* mosaic animals. This contrasts with effects of *Tor* partial loss-of-function mutations, which effectively rescued axon guidance defects of *Tsc1* mutants. Collectively, these findings demonstrate that the role of Tsc-Rheb-Tor signaling in synapse assembly and axon guidance is largely independent of TORC1, S6k, and their effects on growth. Indeed, while animals bearing null alleles of *S6k* have some axon pathfinding defects, the effects are relatively modest compared to *Tsc1* mosaics, indicating that *S6k* does not provide the critical outputs affecting axon guidance.

Our findings parallel recent work in the mouse, where neuronal hypertrophy produced by loss of *Pten* in granule neurons of the cerebellum and dentate gyrus was not rescued by loss of *S6k1* [30]. It is also of note that some but not all *Tsc1/2*-mediated changes in dendritic morphology of hippocampal neurons in organotypic cultures were suppressed by rapamycin treatment [2]. Our findings suggest that inhibition of growth regulatory components in tuberous sclerosis patients, such as achieved with rapamycin and related agents, may not affect all processes that are deranged in the nervous system.

Recent studies of Pi3 kinase, Akt and InR in *Drosophila* have shown that activation of signaling upstream of Tsc1/2 also produces increases in synapse size, both at the NMJ as well as central synapses [31]. Expression of these components in adult neurons demonstrated that Pi3 kinase-mediated synaptogenesis was age-independent, and therefore not a developmentally restricted phenomenon. In agreement with studies reported here, the expanded NMJs produced by activation of Pi3 kinase were functional, with increased stimulus-induced EJPs. Overexpression of the *Drosophila* ortholog of the epidermal growth factor receptor (EgfR) in central neurons increased neuronal cell size, without an increase in synapse number. These results are consistent with those reported here where we have been able to directly suppress growth mediated by Tsc-Rheb-Tor pathway activation without altering effects on synapse formation or axon guidance.

Recent studies have also demonstrated a link between *Tsc1/Tsc2* and *highwire*, a gene known to effect synapse size and functionality in *Drosophila* [32]. The *highwire* ortholog *Pam* was shown to bind Tsc2 in pull-down assays, and it has been suggested that Pam may function as an E3 ubiquitin ligase to regulate the intracellular levels of the Tsc1/Tsc2 complex. This concept of Highwire as a negative regulator of Tsc levels is consistent with our findings, since *highwire* mutants have been shown to possess enlarged NMJs similar to what we see for Rheb overexpression [14]. Despite this, the enlarged synapses of *highwire* mutants display compromised synaptic function which is contrary to what we found when overexpressing Rheb, so Highwire is likely to have multiple functions at the synapse besides simply the regulation of Tsc.

### Contributions of TORC1 versus TORC2 in synapse assembly and axon guidance

Tor has a number of molecular outputs that influence many cellular processes; notable among these are cellular growth and cellular morphology. TORC1, which contains Raptor and is sensitive to the anti-proliferative agent rapamycin, is a major contributor to the regulation of cellular growth, in large measure due to its effects on protein synthesis. TORC2, which includes Rictor, is implicated in the control of cell morphology mediated by regulation of the actin cytoskeleton [33]. Both pharmacological and genetic studies presented here argue in favor of Tor complex 2 providing an essential regulatory component of both synapse growth and axon guidance in *Drosophila*. Our results support recent work showing that changes in dendritic morphology of hippocampal neurons produced by loss of *Tsc1* required regulation of the actin-depolymerizing factor Cofilin [2], implicating TORC2-mediated processes. There is a considerable body of work demonstrating that control of the actin cytoskeleton is critical for NMJ growth and function [34]–[36] and TORC2 may provide an important component of that control. Regulation of actin is also essential for axon guidance in the visual system (reviewed in [37], [38]), and disruption of Tor-mediated control of actin may be the underlying molecular deficit in *Tsc1* mosaics.

### Either gain or loss of Rheb signaling compromises neuromuscular junction assembly and axon guidance

A number of studies have suggested that TOR activation produced by loss of *TSC1/2* affects neuronal morphology and synaptic function. Our findings support these observations; elevated Rheb activity produces synaptic enlargement and enhanced physiological function at the *Drosophila* NMJ. However, it was not evident from earlier studies whether loss of signaling through Rheb and Tor is also important for neural development. We provide evidence that this is the case. Partial loss-of-function mutations in *Rheb* compromise NMJ growth and function, as well as photoreceptor axon targeting in the visual system. Overexpression of *Tsc1* and *Tsc2* in the motoneuron also limited synaptic growth, supporting the conclusion that depressed levels of Rheb activity compromise synapse development.

### Rheb-mediated synaptic development is dependent on a functional BMP signaling system

The capacity of Tsc-Rheb-Tor signaling to affect neuronal morphology and synapse function begs the question of whether these effects are dependent on signaling systems known to be critical for synapse development. At the Drosophila NMJ, BMP signaling is critical for normal growth and function. Mutations in *wit*, a gene encoding a type II BMP receptor, produce a small and poorly functioning NMJ [17], [19]. These deficits can be rescued by motoneuron expression of *wit^+^*, demonstrating that BMP signaling in the motoneuron is critical for synaptic expansion during larval growth. To determine if Rheb-mediated synaptic growth required BMP signaling, we placed *elav-Gal4* and *UAS-Rheb* transgenes into a *wit* mutant background. While overexpression of Rheb and the accompanying activation of the Tor pathway partially rescued the defect in synapse growth produced by loss of *wit* function, it was unable to restore a normal EJP response or rescue quantal content. These findings establish that Tsc-Rheb-Tor mediated effects on synapse morphology are partially dependent on BMP signaling, and are fully dependent on BMP activity for a physiologically competent synapse. Our findings also establish that the functional deficits in *wit* mutants are not simply the result of reduced synapse size, since restoration of synapse size by expression of *UAS-Rheb* does not restore physiological function. Intersection of BMP, and Akt/PTEN/TOR signaling has been noted for other systems, and our results indicate the relationship between these pathways is important for synapse growth and plasticity as well [39].

### Loss of function mutations in *Tsc1* and *Pten* have different effects on axon guidance

Previous analysis of *gigas/Tsc2* mutants demonstrated that loss of this gene in mechanoreceptors affects axon targeting, producing projections to novel areas in the CNS in addition to innervation of normal targets [5]. We have used genetic mosaics to evaluate the function of Tsc-Rheb-Tor signaling in photoreceptor axon guidance. Animals homozygous for *Tsc1* in the retina showed grossly aberrant photoreceptor projections to both the lamina and medulla. R7 and R8 projections to the medulla in 40h pupae failed to terminate correctly and projected beyond normal targets to inappropriate regions within the brain. Somatic mosaics bearing retinal neurons mutant for *Pten* also showed photoreceptor axon guidance defects, but to a notably lesser degree. Since both *Tsc1* and *Pten* alleles used for this analysis were nulls and show comparable effects on cellular growth and differentiation [23], it follows that *Pten* is not as critical for axon guidance as *Tsc1*. The distinctions between axon guidance phenotypes of *Pten* and *Tsc1* null mutants indicate that altered timing of differentiation is not critical for axon guidance and that control of this pathway at the level of *Pten* or *Tsc1* is not functionally equivalent. Our findings that rapamycin arrests retinal overgrowth produced by loss of *Tsc1* but not *Pten* in the retina supports earlier work demonstrating that retinal overgrowth mediated by loss of *Tsc1*, but not *Pten*, can be suppressed by reductions in *S6k* activity [40]. Those results were interpreted as demonstrating that Pten is largely a regulator of Akt activity, whereas Tsc1/2 serves as a tumor suppressor and inhibitor affecting principally S6k. Our results support these relationships and emphasize that in the nervous system regulation of Tsc1/2 targets other than S6k are critical.

### Graded activation of the Tsc-Rheb-Tor signaling axis produces graded effects on axon guidance

We have used two different genetic methods for activating the Tsc-Rheb-Tor pathway in the visual system; generating retinal mosaics with a loss of function allele of *Tsc1*, and pan-neuronal expression of Rheb using *elav-Gal4* and *UAS-Rheb*. The comparison of these methods revealed that overexpression of *Rheb* produced milder axon guidance phenotypes in the visual system than complete loss of *Tsc1* function. Of interest is that the degree of activation achieved with *elav-Gal4>UAS-Rheb*, a level that did not produce lethality, did result in discernable axon targeting defects in the visual system. This suggests that axon guidance controlled by Tsc-Rheb-Tor is sensitive to incremental changes in signaling. The range of neurological and behavioral phenotypes associated with loss of one copy of *TSC1* or *TSC2* is consistent with this model, where other environmental or genetic factors may affect signaling levels, producing a range of deficits. Our findings indicate that *Drosophila* can serve as a useful model for identifying how graded changes in signaling can produce a spectrum of defects in neural development.

## Materials and Methods

### Drosophila strains

*UAS-Rheb^EP50.084^/TM6B Tb* [11], *UAS-DP110^WT^* [41], and *y w hsFLP; UAS-Tsc1 UAS-Tsc2* [21] were crossed to *elav-Gal4/CyO P[w^+^; ubi-GFP]* [42] or *OK6-Gal4* [17] for expression in neurons, and to *G14-Gal4/CyO P[w^+^; ubi-GFP]* (from C. Goodman) for expression in muscles. Stocks used for mutant analysis and genetic interaction studies were *y w; Rheb^3M2^/TM6B Tb y^+^* [11], *Rheb^2D1^/TM6B Tb* [11], *b w; wit^B11^/TM6B Tb* [19], *w; wit^A12^/TM6B Tb* [19], *S6k^l-1^/TM6B Tb* [43], and *Tor^2.1/Ala948Val^/CyO P[w^+^; ubi-GFP]* [44]. Eye-specific mosaics were generated using the *FLP-FRT* technique [45] by crossing *y w eyFLP GMR-lacZ; FRT82B I(3)cl-R3/TM6B Tb* [45] or *y w eyFLP GMR-lacZ; I(2)cl-L3 w^+^ FRT40A/CyO y^+^* [45] to *w; FRT82B Tsc1^29^/TM6B Tb* [20], *w; FRT82B Rheb^26.2^/TM6B Tb y^+^* [11], *y w hsFLP; FRT40A Pten^dj189^/SM6-TM6B* [24], *y w eyFLP GMR lacZ; FRT82B* [45], or *y w eyFLP GMR lacZ; FRT40A* [45]. Using this mosaic method, heterozygous cells have a growth disadvantage since they bear a *Minute* and cell-lethal mutation [*l(3)cl-R3* or *l(2)cl-L3*]. For all retinal mosaics, we assessed the degree of mosaicism by examining the adult retina where mutant cells could be identified by loss of the *w+* marker. In *Tsc1, Pten* and *Rheb* mosaics, mutant cells comprised the vast majority of the adult retina (>90%). Wild-type strains were y w, Oregon-R, or Canton-S.

### Immunohistochemistry

For visualization of neuromuscular junction synapses, third instar larvae were filleted in PBS and fixed in 4% formaldehyde before staining with Anti-Cysteine String Protein mAB49 at 1∶1000 (a generous gift from Zinsmaier and Buchner) and FITC-conjugated Anti-HRP at 1∶50 (Jackson Labs). Bouton numbers were determined by a combination of CSP and HRP image data. Muscle surface area measurements were performed using ImageJ data analysis software (NIH) and represent the combined area of the second abdominal segment muscles 6 and 7. Third instar larvae and 40hr pupae were fixed, stained, and mounted as described [46] for photoreceptor analysis. Antibodies from the Developmental Studies Hybridoma Bank were used at 1∶25 for mouse anti-Chaoptin (MAb24B10), 1∶10 for mouse anti-Repo (MAb8D12), and 1∶25 for mouse anti-Dachshund (MabDac2-3). Secondary antibodies were from the AlexaFluor series (Invitrogen). Texas Red-phalloidin was used at 0.165 µM (Invitrogen). All images were acquired on a Nikon C1 upright laser confocal.

### Rapamycin Treatment

Flies were raised on standard laboratory food supplemented with rapamycin (Sigma) to a final concentration of 3 µM for NMJ analysis or 2 µM for eye disks. Rapamycin treated *Tsc1^29^* mosaic animals with eye discs similar in size to control animals were selected for photoreceptor projection analysis.

### Electrophysiology

Excitatory junctional potential (EJP) recordings were taken from muscle 6 in the second abdominal hemisegment of 3^rd^ instar larvae. Dissections were done in Ca^++^-free saline and recordings were performed in HL3 following published protocols [47]. Recordings were acquired with an Axoclamp 2B amplifier and pClamp9 software (Axon Instruments). EJP amplitudes and mini-EJP amplitudes were measured with MiniAnalysis software from Synaptosoft.

## Supporting Information

Figure S1 Patterning of lamina precursor cells and glia in *Pten* or *Tsc1* mosaic animals. (A–F) Dorsal-posterior views of third instar larval optic lobes stained with anti-Dachshund (lamina precursor cell marker) or anti-Repo (glial cell marker). (A–C) *Pten* mosaic animals show a significantly larger lamina compared to control animals. This is not seen to the same extent in *Tsc1* mosaics. (D–F) Glial cells successfully differentiate and migrate in both *Pten* and *Tsc1* mosaics, however mild patterning defects are apparent and could possibly contribute to the photoreceptor patterning abnormalities observed. All scale bars are 50 microns.
(1.32 MB TIF)
