# Supplementary figures and images for: Mechanisms of TSC-mediated Control of Synapse Assembly and Axon Guidance
Source: PLoS One. 2007 Apr 18;2(4):e375. doi: 10.1371/journal.pone.0000375 (PMC1847706; doi:10.1371/journal.pone.0000375)

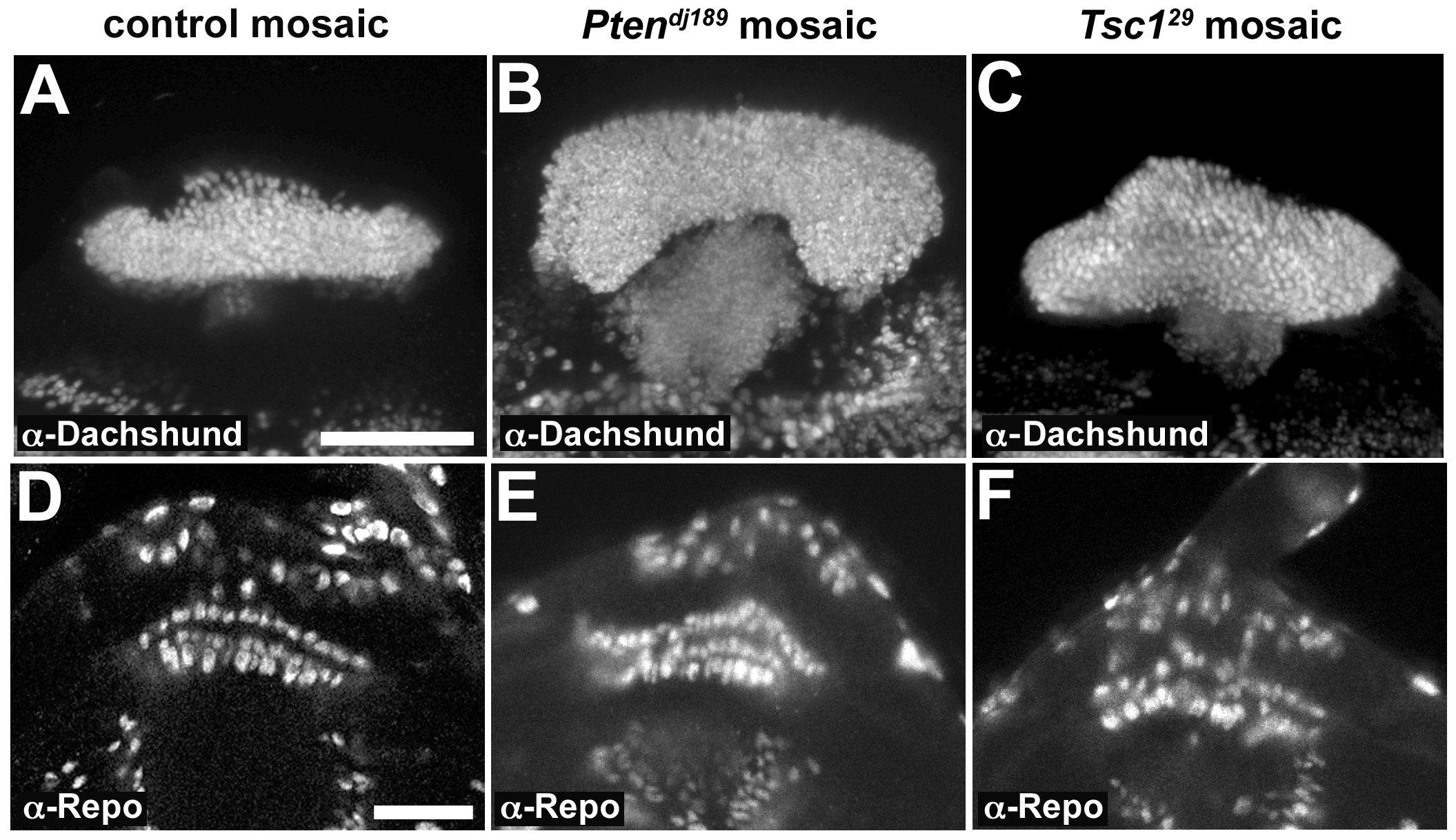

Supplement: Figure S1 — Patterning of lamina precursor cells and glia in Pten or Tsc1 mosaic animals. (A–F) Dorsal-posterior views of third instar larval optic lobes stained with anti-Dachshund (lamina precursor cell marker) or anti-Repo (glial cell marker). (A–C) Pten mosaic animals show a significantly larger lamina compared to control animals. This is not seen to the same extent in Tsc1 mosaics. (D–F) Glial cells successfully differentiate and migrate in both Pten and Tsc1 mosaics, however mild patterning defects are apparent and could possibly contribute to the photoreceptor patterning abnormalities observed. All scale bars are 50 microns. (1.32 MB TIF) [file pone.0000375.s001.tif]
